# MRI-Based Brain Volumetry at a Single Time Point Complements Clinical Evaluation of Patients With Multiple Sclerosis in an Outpatient Setting

**DOI:** 10.3389/fneur.2018.00545

**Published:** 2018-07-25

**Authors:** Alaleh Raji, Ann-Christin Ostwaldt, Roland Opfer, Per Suppa, Lothar Spies, Gerhard Winkler

**Affiliations:** ^1^Neurozentrum Hamburg, Hamburg, Germany; ^2^jung diagnostics GmbH, Hamburg, Germany

**Keywords:** multiple sclerosis, magnetic resonance imaging, brain atrophy, thalamus, volumetry

## Abstract

**Purpose:** Thalamic atrophy and whole brain atrophy in multiple sclerosis (MS) are associated with disease progression. The motivation of this study was to propose and evaluate a new grouping scheme which is based on MS patients' whole brain and thalamus volumes measured on MRI at a single time point.

**Methods:** In total, 185 MS patients (128 relapsing-remitting (RRMS) and 57 secondary-progressive MS (SPMS) patients) were included from an outpatient facility. Whole brain parenchyma (BP) and regional brain volumes were derived from single time point MRI T1 images. Standard scores (z-scores) were computed by comparing individual brain volumes against corresponding volumes from healthy controls. A z-score cut-off of −1.96 was applied to separate pathologically atrophic from normal brain volumes for thalamus and whole BP (accepting a 2.5% error probability). Subgroup differences with respect to the Symbol Digit Modalities Test (SDMT) and the Expanded Disability Status Scale (EDSS) were assessed.

**Results:** Except for two, all MS patients showed either no atrophy (group 0: 61 RRMS patients, 10 SPMS patients); thalamic but no BP atrophy (group 1: 37 RRMS patients; 18 SPMS patients) or thalamic and BP atrophy (group 2: 28 RRMS patients; 29 SPMS patients). RRMS patients without atrophy and RRMS patients with thalamic atrophy did not differ in EDSS, however, patients with thalamus and BP atrophy showed significantly higher EDSS scores than patients in the other groups.

**Conclusion:** MRI-based brain volumetry at a single time point is able to reliably distinguish MS patients with isolated thalamus atrophy (group 1) from those without brain atrophy (group 0). MS patients with isolated thalamus atrophy might be at risk for the development of widespread atrophy and disease progression. Since RRMS patients in group 0 and 1 are clinically not distinguishable, the proposed grouping may aid identification of RRMS patients at risk of disease progression and thus complement clinical evaluation in the routine patient care.

## Introduction

Next to lesion burden as a marker for disease activity, brain atrophy measured by magnetic resonance imaging (MRI) has in recent years been established as an important biomarker of tissue damage and neurodegeneration in multiple sclerosis (MS) (1–4). The approaches to measure brain atrophy in MS are not standardized yet and potentially influenced by biological and technical parameters (1, 5). However, several studies showed an association of whole brain atrophy with worsening of the Expanded Disability Status Scale (EDSS) as a measure of physical disability (6–8). A recent review summarizes that brain atrophy in both cross-sectional and longitudinal studies predicts disability progression and is associated with cognitive impairment (1). Consequently, brain atrophy has been a target for multiple pharmacological phase II and III studies (1, 5).

Besides whole brain atrophy, the volume of subcortical structures, like putamen (9, 10) and caudate nucleus (10), as well as the corpus callosum (11, 12) were found to be reduced in MS. The thalamus has been shown to be especially susceptible to atrophy already at early stages of MS (10, 13, 14), and early thalamic atrophy has even been discussed as a marker to predict the transition from clinically isolated syndrome (CIS) to clinically definite MS (15). Several longitudinal (16, 17) studies as well as a cross-sectional study (14) showed that thalamic atrophy is associated with progression of disability in MS patients, while some other studies found only a weak (10) or no association between thalamus volume and the EDSS (18). A reduction in volume and connectivity in the thalamus was also found to be associated with cognitive decline (19–21), and fatigue (22). Taken together, measures of both whole brain and regional subcortical thalamus atrophy could help to predict a worsening of clinical symptoms in individual MS patients.

In most studies, brain atrophy is measured as brain volume change between two examination time points. The interval between examinations must be sufficiently large, in order to reliably detect significant atrophy. In the present study, MRI-based brain volumetry was applied to MRI scans of a cohort of MS patients from an outpatient facility reflecting a typical clinical distribution of different MS phenotypes. The motivation of this study was to show that MRI-based brain volumetry at a single time point is able to reliably identify MS patients with an isolated thalamic atrophy and thus may complement the clinical evaluation of MS patients. This subgroup of patients with an isolated thalamic atrophy might be of special clinical interest, since thalamic atrophy might mark the beginning of a more pronounced whole brain atrophy which in turn is associated with physical and cognitive decline (13, 14, 16, 17, 19–21). Therefore, whole brain parenchyma (BP) and thalamic volumes of MS patients measured at a single time point were compared against corresponding volumes of a cohort of healthy control subjects. Based on these comparisons MS patients were grouped according to the presence or absence of thalamic and BP atrophy. Since previous studies showed that thalamic atrophy is pronounced in MS and might occur earlier than BP atrophy (10, 13, 14, 23), it was expected that no patients with BP atrophy but without thalamic atrophy can be found in this cohort of MS patients. In contrast, it was expected that a substantial proportion of included MS patients is characterized by an isolated thalamic atrophy with a non-measurable whole brain atrophy.

## Patients and methods

### Patient cohort and healthy control subjects

Patients with definite relapsing-remitting (RRMS) or secondary-progressive MS (SPMS) were included upon first visit at an outpatient facility specialized to MS (Neurozentrum Hamburg, Germany). Exclusion criteria were MRI acquisition with deviating sequence parameters or strong MRI artifacts and a primary progressive disease course. This study was carried out in accordance with the recommendations of the Ärztekammer Hamburg with written informed consent from all subjects. All subjects gave written informed consent in accordance with the Declaration of Helsinki. The protocol was approved by the Ärztekammer Hamburg.

The EDSS (24) was assessed for each patient by an experienced neurologist. The EDSS measures disability in eight functional systems, and the results can range from 0 (normal neurological exam) to 10 (death due to MS). The Symbol Digit Modalities Test [SDMT (25)] was assessed as a measure of cognitive impairment. The SDMT is a standardized score, meaning that an average performance of a healthy subject is denoted by an SDMT score of 0, reduced cognitive performance would reflect in a negative SDMT score. Disease duration was estimated as time since first symptoms were documented.

Subjects without clinical symptoms as confirmed by an experienced neurologist and without evidence of pathological brain abnormalities on the MRI scan as confirmed by an experienced neuroradiologist were included as healthy control subjects.

### MRI acquisition

MRI brain scans of MS patients and healthy control subjects were acquired in the clinical routine at two different radiological facilities. One radiological facility was equipped with a GE Signa 3 Tesla (T) scanner system (General Electric, Milwaukee, WI, USA) whereas the other radiological facility was equipped with a Philips Achieva 3 T scanner system (Philips Healthcare, Eindhoven, the Netherlands).

Each MRI examination included the acquisition of a high resolution pre-contrast 3D T1-weighted sequence and a Fluid Attenuated Inversion Recovery (FLAIR) sequence. On the GE scanner system the T1 was acquired using a 3D Fast Spoiled Gradient Echo (FSPGR) sequence [repetition time (TR) = 6.8 ms, echo time (TE) = 1.9 ms, inversion time (TI) = 450 ms, voxel size = 1.02 × 1.02 × 1 mm, scan time = 4:47 min] and the FLAIR sequence was acquired using a 3D CUBE sequence (TR = 8,000 ms, TE = 85–127 ms, TI = 2,123–2,296 ms, voxel size = 0.51 × 0.51 × 2.6 mm, scan time = 4:43 min). On the Philips scanner system the T1 was acquired using a 3D Turbo Field Echo (TFE) sequence (TR = 8.1 ms, TE = 3.7 ms, voxel size = 0.86 × 0.86 × 1 mm, scan time = 3:37 min) and the FLAIR sequence was acquired using a 3D Volume Isotropic Turbo spin echo Acquisition (VISTA) sequence (TR = 8,000 ms, TE = 337 ms, TI = 2,400 ms, voxel size = 0.44 × 0.44 × 2 mm, scan time = 4:43 min). The exact same acquisition protocol settings were applied to acquire the brain scans of both MS patients and healthy control subjects.

### MRI-based brain volumetry

MRI scans were processed using the Biometrica MS® analysis platform (version 2.1, jung diagnostics GmbH, Hamburg, Germany). The first step was the automated estimation of the T2 hyperintense lesion load using the Lesion Segmentation Tool (LST) which is based on the Statistical Parametric Mapping (SPM8, version 8, http://www.fil.ion.ucl.ac.uk/spm/software/spm8/) software package. The resulting lesion map image was used to calculate the FLAIR lesion load (in ml). Furthermore, the lesion map was used to replace the voxel intensities of lesion voxels in the T1-weighted MRI image with estimated healthy white matter intensity. The rationale for this step was to mitigate the effect of WM hyperintensities on automatic brain tissue segmentation as described in detail elsewhere (26). The lesion-corrected T1 MRI images were segmented using a previously described and validated atlas-based volumetry approach implemented in SPM12 (27, 28). In brief, MRI brain scans were segmented into tissue class component images representing either gray matter (GM), WM or cerebrospinal fluid (CSF). GM and WM volumes were derived by summing up the voxel intensities within each component image. Global BP volume was defined as the sum of GM and WM volume. Regional brain volumes were derived by the multiplication of the tissue class component images with a predefined binary mask and subsequent integration of voxel intensities within the resulting masked image. The thalamus mask was taken from the Wake Forest University Pickatlas (29), http://fmri.wfubmc.edu/software/pickatlas). Masks for the caudate nucleus and putamen were taken from the LPBA40 atlas (30). The corpus callosum mask contained binary masks from subregions, i.e., genu, body, and splenium, taken from the ICBM-DTI-81 white matter labels atlas (31, 32). Volumes of the thalamus and the putamen were defined as the sum of GM and WM volume within the respective brain region. The volume of the caudate nucleus was obtained from the GM component image only and the volume of the corpus callosum was defined from the WM component image only. The total intracranial volume (TIV) was estimated using a method which was recently introduced and validated by Malone et al. (33). Results of the lesion and tissue segmentation were visually checked for segmentation errors.

### Adjustment of brain volumes for head-size and age

Individual brain volumes were adjusted for head-size and age to minimize the impact of these confounding variables on statistical analysis. TIV was used as a surrogate measure for head-size. The adjustment was performed by computing the residuals from a linear regression function. The linear regression function was estimated based on brain volumes obtained from the T1-weighted MR images of a cohort of 316 healthy control subjects provided by the Open Access Series of Imaging Studies [OASIS, (34)]. Brain volumes of healthy control subjects were derived by using the Biometrica MS® platform as described in section MRI-based Brain Volumetry. Furthermore, a non-linear regression technique was applied to the brain volumes of the 316 healthy control subjects from OASIS to derive the age-volume trajectory of physiological aging as described recently (35). Subsequently, individual TIV-adjusted brain volumes were adjusted for age by computing the residuals with respect to the estimated age-volume trajectories.

### Standard score (Z-score) calculation

For each scanner system (GE Signa 3T and Philips Achieva 3 T), head-size- and age-adjusted brain volumes of MS patients were compared to the adjusted brain volumes of the corresponding healthy control subjects whose MRI scans were acquired on the same MRI scanner system using the same acquisition protocol settings. For each MS patient, a z-score was calculated according to the formula $\frac{v_{i}-mean\left(controls\right)}{std\left(controls\right)}$, where *v*_*i*_ denotes the patient's brain volume adjusted for head-size and age, *mean*(*controls*) and *std*(*controls*) denote the mean and the standard deviation of the corresponding cohort of healthy control subjects from the respective scanner system. With the calculation of z-scores, we take the variable “scanner” into account for the statistical analyses: The calculation of z-scores enables pooling and direct comparison of brain volumes derived from both scanner systems.

### Cut-off value and grouping of MS patients

Assuming a normal distribution in the cohort of healthy control subjects, 95% of the brain volume values are located within the area of the mean ± 1.96^*^standard deviation. Only 5% of the brain volume values are expected to be larger or smaller. Therefore it can be assumed that z-scores below −1.96 represent a significant brain volume reduction with an error probability of 2.5% at most. The z-score cut-off of −1.96 was consequently applied to group individual MS patients based on their BP and thalamus volumes into the following subgroups:

Group 0: no thalamic or BP atrophy (i.e., both within the normal range; z-scores greater than −1.96)
Group 1: thalamic (z-score below −1.96) but no BP atrophy (z-score greater than −1.96)
Group 2: thalamic and BP atrophy (both z-scores below −1.96).

### Statistics

Descriptive statistics were reported for the whole patient cohort and for the subgroups of RRMS and SPMS patients separately. Differences between RRMS and SPMS patients were tested for significance using the Wilcoxon rank sum test.

The distribution of z-scores of the different brain regions was tested for a significantly different deviation from a distribution with a zero mean using a one-sample *t*-test. Differences in z-scores between different brain regions were tested with an analysis of variance (ANOVA) and a Bonferroni *post-hoc* test. After grouping of MS patients as described in the previous section, various clinical parameters were tested for between-group differences using the Kruskal-Wallis test. In case of a significant between-group difference, a Dunn and Sidák's *post-hoc* test was applied for pairwise comparison.

A *p*-value below 0.05 was a priori defined as significant. Statistical analyses were performed using MATLAB (R2014b, The Mathworks Inc., Natick, USA) and its Statistics and Machine Learning toolbox.

## Results

### Patient characteristics

In total, 185 MS patients were included at their first visit at the Neurozentrum Hamburg. Of these, 55 patients were scanned on the Philips Achieva 3 T scanner system and 130 MS patients were scanned on the GE Signa 3 T scanner system. Furthermore, 84 healthy control subjects were included (50 subjects were scanned on the Philips Achieva 3 T and 34 subjects on the GE Signa 3 T scanner system). Characteristics and volumetric results for patients and healthy control subjects separated according to the MRI scanner system used for MRI acquisition can be found in Supplementary Table 1. Clinical characteristics of the whole patient cohort and for the clinical subgroups of RRMS and SPMS patients are summarized in Table 1. RRMS patients were found to be significantly younger, were characterized by a shorter disease duration, a lower EDSS score, and a higher SDMT score compared to SPMS patients (all *p* < 0.001).

**Table 1:** Patient characteristics and volumetric results for the whole MS patient cohort.

	**All MS patients**	**RRMS patients**	**SPMS patients**	***p*-value**
Sample size	185	128	57	
Females	133 (71.9%)	94 (73.4%)	39 (68.4%)	
Age (years)	43.5 (33.1; 50.3)	39.8 (30.3; 46.0)	51.6 (45.8; 61.1)	< 0.0001
Disease duration (years)	7 (2.4; 14)	5 (2; 9)	16 (11; 22.8)	< 0.0001
EDSS	2 (1; 4.5)	1.5 (1; 2)	6 (5; 7)	< 0.0001
SDMT	−1 (−2; 0)	−0.5 (−1; 0)	−1.75 (−2.5; −1)	< 0.0001
Treatment				
Yes	108 (58.4%)	84 (65.6%)	24 (42.1%)	
No	77 (41.6%)	44 (34.4%)	33 (57.9%)	
BP volume z-score	−1.13 (−2.50; −0.16)	−0.81 (−1.92; 0.12)	−2.14 (−3.43; −0.94)	< 0.0001
GM volume z-score	−0.76 (−1.57; 0.13)	−0.60 (−1.28; 0.37)	−0.93 (−1.86; −0.15)	0.0058
WM volume z-score	−1.02 (−2.06; −0.09)	−0.81 (−1.58; 0.00)	−1.82 (−2.94; 0.71)	< 0.0001
Corpus callosum volume z-score	−1.50 (−2.87; −0.52)	−1.21 (−2.31; 0.28)	−2.52 (−3.94; −1.34)	< 0.0001
Caudate nucleus volume z-score	−0.89 (−1.87; 0.10)	−0.64 (−1.49; 0.12)	−1.57 (−2.39; 0.76)	< 0.0001
Thalamus volume z-score	−2.46 (−3.99; −0.96)	−1.97 (−3.24; −0.63)	−3.50 (−5.14; −2.48)	< 0.0001
Putamen volume z-score	−1.20 (−2.45; −0.25)	−0.91 (−1.76; −0.12)	−2.11 (−3.10; 0.79)	< 0.0001

*Z-scores were calculated in comparison to the respective scanner-specific control cohort. All values are given as median and interquartile range or frequency and percentage. Symbol Digit Modalities Test (SDMT) was assessed for 183 of the 185 patients. Values are also given for the groups of relapsing-remitting MS (RRMS) and secondary-progressive MS (SPMS) patients separately. P-values indicate the difference between RRMS and SPMS patients, calculated with the Wilcoxon rank sum test. EDSS, Extended disability status scale; BP, brain parenchyma; GM, gray matter; WM, white matter*.

### Brain volumes

**Figure 1:**
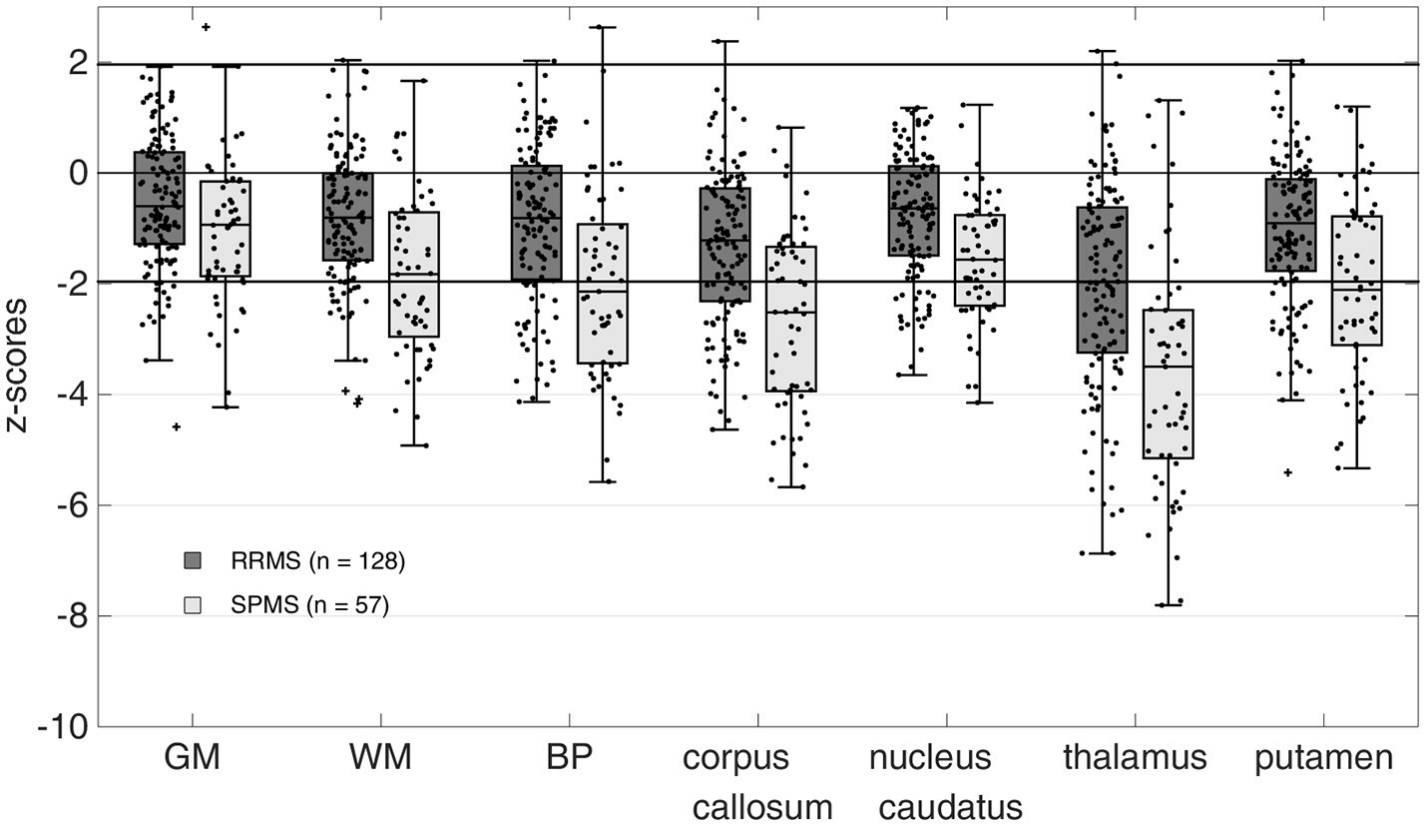
Z-scores of calculated brain volumes. Brain volumes were normalized to the respective scanner-specific control cohort. Z-Scores for all regions were significantly different from zero (*p* < 0.0001 for all brain regions). Mean z-scores for the thalamus were significantly lower than all other brain volumes (*p* = 0.0295 for comparison with corpus callosum, *p* < 0.001 for all other comparisons, ANOVA with Bonferroni correction) in both the relapsing-remitting MS (RRMS) and secondary progressive MS (SPMS) patients. GM, gray matter; WM, white matter; BP, brain parenchyma.

The distribution of z-scores for BP and regional brain volumes of RRMS and SPMS patients are summarized in Table 1 and additionally illustrated in Figure 1. Median z-scores for all brain regions were significantly lower than zero (*p* < 0.0001 for all brain regions) and generally lower for SPMS compared to RRMS patients. The median z-score for the thalamus volume was significantly lower compared to all other brain regions in both RRMS and SPMS patients (*p* = 0.0295 for comparison with corpus callosum, *p* < 0.001 for all other comparisons).

### Grouping of the MS patients according to thalamus and BP atrophy

**Figure 2:**
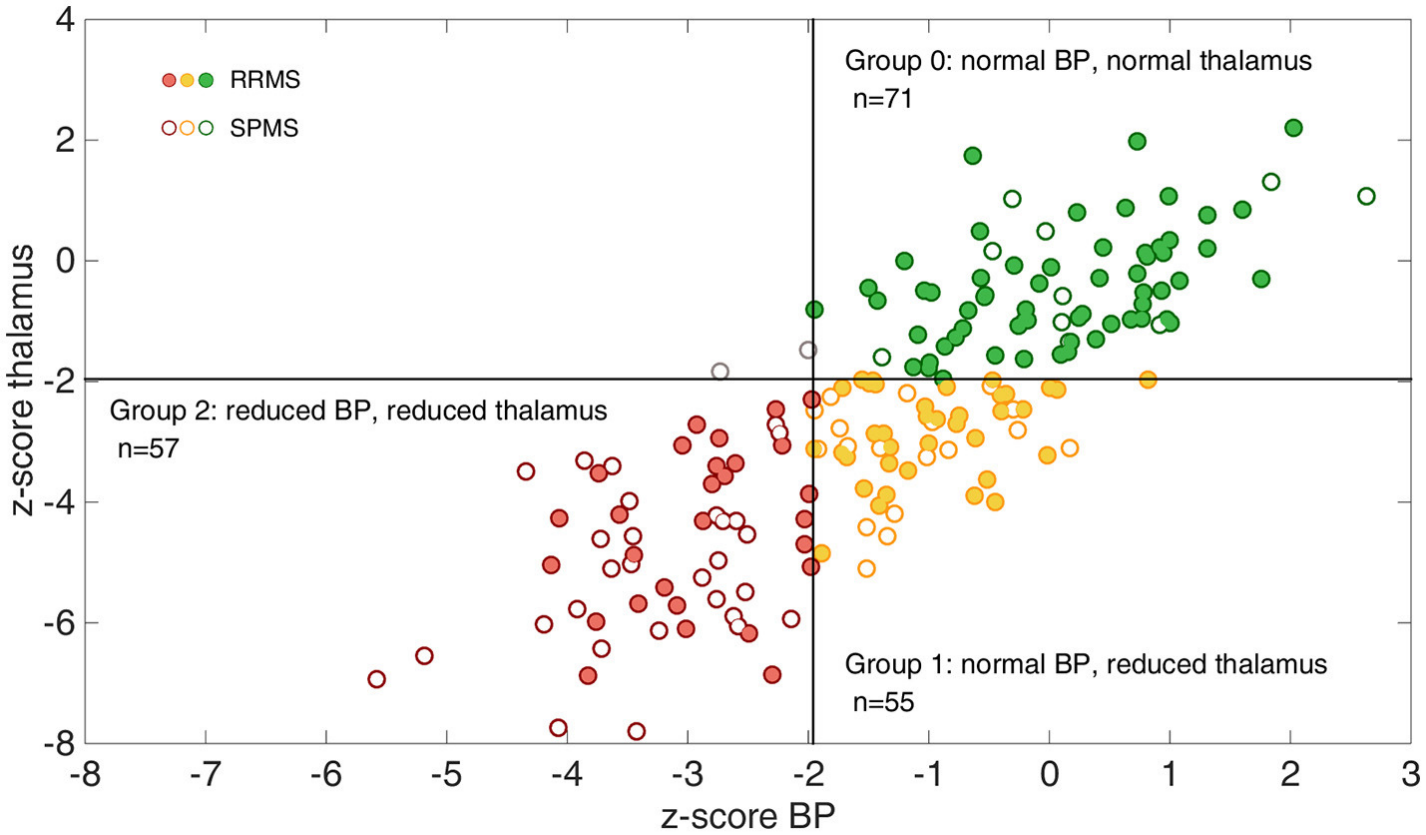
Illustration of the grouping of the whole MS patient cohort according to thalamic and whole brain atrophy. The association between z-scores for brain parenchyma (BP) and z-scores for thalamus volumes are shown. Using a cut-off of −1.96 almost all patients (except for two) fall into one of the three groups: group 0 = no atrophy (green circle, upper right quadrant), group 1 = thalamic atrophy without BP atrophy (yellow circle, lower right quadrant) and group 2 = thalamic and BP atrophy (red circle, lower left quadrant). Relapsing-remitting MS (RRMS) patients are marked with a filled circle in all groups, while secondary-progressive MS (SPMS) patients are marked with open circles.

The distribution of MS patients across the three subgroups based on the z-score cut-off of −1.96 for regional thalamic and BP atrophy is shown in Figure 2. Almost all MS patients fit in one of the following three groups: 71 MS patients (38%, 61 RRMS, 10 SPMS) showed no thalamic or BP atrophy, 55 MS patients (30%, 37 RRMS, 18 SPMS) showed thalamic atrophy but no BP atrophy and 57 MS patients (31%, 28 RRMS, 29 SPMS) showed both, thalamic and BP atrophy. Only two patients with BP atrophy but without thalamic atrophy were found. These MS patients were omitted from further statistical analyses.

### Association of grouping and clinical parameters

Clinical characteristics for the three subgroups, separated by RRMS and SPMS patients, are summarized in Table 2. The number of RRMS patients in each subgroup decreased with increasing atrophy (i.e., from the subgroup without atrophy to the subgroup with thalamic and BP atrophy). In contrast, the number of SPMS patients in each subgroup increases with increasing atrophy. RRMS patients with both thalamic and BP atrophy had a significantly longer disease duration compared to RRMS patients without atrophy (*p* = 0.036).

**Table 2:** Characteristics of the RRMS and SPMS patients, grouped according to atrophy in thalamus and brain parenchyma (BP).

	**Group 0** ** (no atrophy)**		**Group 1** ** (thalamus atrophy only)**		**Group 2** ** (thalamus and BP atrophy)**		***p*****-value** ** Kruskal-Wallis**	
	**RRMS**	**SPMS**	**RRMS**	**SPMS**	**RRMS**	**SPMS**	**RRMS**	**SPMS**
*n*	61	10	37	18	28	29		
Thalamus volume (z-score)	−0.57 (−1.05; 0.09)	−0.21 (−1.06; 1.03)	−2.71 (−3.28; −2.13)	−3.09 (−3.25; −2.48)	−4.27 (−5.55; −3.38)	−5.10 (−6.03; −4.29)		
BP volume (z-score)	0.15 (−0.64; 0.79)	−0.11 (−0.31; 0.91)	−1.02 (−1.46; −0.47)	−1.31 (−1.67; −0.84)	−2.84 (−3.43; −2.28)	−3.43 (−3.76; −2.61)		
FLAIR lesion load (ml)	1.0 (0.5; 2.9)	4.1 (1.6; 8.8)	3.8 (1.3; 8.0)	11.6 (5.6; 28.8)	11.2 (4.4; 28.4)	24.3 (20.4; 31.6)	< 0.001^*^^^+^^♢^^	0.001^*^
disease duration (years)	4 (1; 7)	15 (10; 30)	5 (2; 8.5)	17 (11; 21)	7 (4; 11)	15 (12; 21)	0.0386^*^	0.987
EDSS score	1.5 (1.0; 1.6)	5.5 (5.0; 6.5)	1.5 (1.0; 2.0)	6.0 (4.0; 6.5)	2.0 (1.5; 2.5)	6.0 (5.0; 7.0)	0.001^*^^^+^^	0.249
SDMT score	−0.5 (−1.0; 0)	−1.0 (−2.0; 0)	−0.5 (−1.0; 0)	−1.5 (−2.0; −1.0)	−1.0 (−2.0; 0)	−2.0 (−3.0; −1.3)	0.368	0.032^*^

*Values are given as median and interquartile range. Symbol Digit Modalities Test (SDMT) was assessed for 181 of the 183 categorized patients. P-values indicate the difference between group 0, 1 and 2, both for the relapsing-remitting MS (RRMS) and secondary progressive MS (SPMS) patients separately. FLAIR, fluid attenuated inversion recovery; EDSS, Extended Disability Status Scale*.

* group 2 significantly different from group 0 (post-hoc Dunn-Sidak test).

+ *group 2 significantly different from group 1 (post-hoc Dunn-Sidak test)*.

**#x02662;:** *group 0 significantly different from group 1 (post-hoc Dunn-Sidak test)*.

The EDSS score for the subgroup of RRMS patients with thalamic and BP atrophy was significantly higher compared to RRMS patients without atrophy (*p* < 0.001) and those with an isolated thalamus atrophy (*p* = 0.01). No significant difference between RRMS patients without and with isolated thalamus atrophy was found with respect to the EDSS (*p* > 0.05). Within the SPMS patients, no significant difference with respect to disease duration or EDSS was found between the atrophy subgroups. A significantly lower SDMT score was found for SPMS patients with thalamic and BP atrophy compared to those without atrophy (*p* = 0.047).

A significantly higher FLAIR lesion load was found for RRMS patients with thalamic and BP atrophy compared to RRMS patients without and those with an isolated thalamus atrophy (*p* < 0.001 for both comparisons). For SPMS patients a significantly higher FLAIR lesion load was only found for patients with thalamic and BP atrophy compared to SPMS patients without atrophy (*p* = 0.002).

### Grouping of the MS patients according to other subcortical volumes

To emphasize the unique role of the thalamus, we additionally performed the grouping by replacing the thalamus volume with the volume of the corpus callosum. Within this setup, 11 MS patients were found with BP atrophy but no corpus callosum atrophy. Moreover, the z-score values were found to be more evenly distributed within each of the subgroups (see Supplementary Figure 1). Similar results were obtained when putamen and the caudate nucleus volumes were used as an early indicator of regional atrophy in MS (results not shown).

## Discussion

In this study, MRI-based brain volumetry at a single time point was applied to MS patients from clinical routine patient care to evaluate its potential to complement clinical assessment in identifying MS patients at risk of disease progression. The proposed grouping scheme created subgroups comprising MS patients without thalamic and BP atrophy (group 0), those with an isolated thalamic atrophy (group 1) and those with a pronounced thalamic and BP atrophy (group 2). Two out of 185 MS patients showed BP atrophy and no thalamic atrophy according to the predefined z-score cut-off of −1.96. However, the z-score values for the thalamus and BP volume (thalamus: z-score = −1.48 and −1.83 and BP: z-score = −2.0 and −2.7 for the first and second outlier patient, respectively) were found to be close to the cut-off and thus might be considered to reflect the uncertainty of the applied cut-off value. These results therefore confirm our hypothesis, that hardly any patients with BP atrophy but without thalamic atrophy are present in this cohort of MS patients. Furthermore, we were able to confirm the hypothesis that a substantial proportion of MS patients is characterized by an isolated thalamic atrophy with a non-measurable whole brain atrophy. A significant atrophy in the thalamus was expected, since previous studies demonstrated that thalamic atrophy is pronounced even at early disease stages (10, 14, 23, 36). However, only a few studies so far suggested that subcortical atrophy might precede whole brain atrophy in MS (10, 14). The results of our study demonstrate with a different segmentation technique and a pre-defined cut-off that in a subset of patients, thalamus atrophy without whole brain atrophy can be shown. But even more interesting, we were able to show that almost no patients show whole brain atrophy, without thalamus atrophy. This seems to be a unique feature of the thalamus and cannot be reproduced if other subcortical brain volumes are used for grouping (see Supplementary Figure 1). A possible explanation is that corpus callosum, putamen and the caudate nucleus show a lower susceptibility for atrophic processes compared with the thalamus (see Figure 1). This indicates that the atrophic process in these regions might advance rather simultaneously with whole brain volume loss.

Based on EDSS and SDMT, RRMS patients in group 0 and 1 were clinically not distinguishable (*p* > 0.05). Thus, thalamus atrophy in group 1 is an important subclinical finding. Not until further progression in terms of additional BP atrophy (group 2), a significant difference with higher EDSS in group 2 compared with group 1 (*p* = 0.01) develops. Thus, there is a strong indication that RRMS patients with an isolated thalamic atrophy might be at a transitional stage toward the development of a widespread whole brain atrophy and an accompanied disease progression. This grouping of RRMS patients based on a single time point MRI might support the clinical evaluation of MS patients and contribute to the selection of disease-modifying drugs. However, the prognostic value of the proposed cut-offs for brain atrophy concerning disease progression needs to be evaluated in longitudinal studies. The present study therefore serves as a proof of concept study.

Included SPMS patients were generally characterized by a higher EDSS score compared to RRMS patients. Whereas RRMS patients showed EDSS scores between 1.5 and 2 indicating a functional impairment only, the EDSS score in SPMS patients ranged between 5.5 and 6 meaning that SPMS patients are already disabled but still able to carry out activities of daily living by themselves (37). Although no significant difference with respect to the EDSS score between subgroups of SPMS patients was found, there might be differences with respect to the performance in functional systems in these groups of SPMS patients. This assumption is supported by the significantly lower SDMT score in SPMS patients with a thalamic and BP atrophy compared to SPMS patients without atrophy (*p* < 0.05). Therefore, brain volume assessment using MRI-brain volumetry at a single time point might also be helpful for the clinician to monitor SPMS patients and to take the neurodegenerative component of MS into account.

The amount of lesion load in the included MS cohort increases in parallel to the amount of brain atrophy in both RRMS and SPMS patients. This result point to a direct relationship between lesion load and brain atrophy, however, the discussion whether inflammation and atrophy are two independent disease processes in MS is still ongoing (38).

Overall, our results indicate an individual course of disease as the severity of atrophy might not be directly linked to disease duration. For instance, 10 SPMS patients with a median disease duration of 15 years and a median EDSS score of 5.5, but without evidence of atrophy in the thalamus or BP were found. A possible explanation for this finding is an increased amount of lesions in the spinal cord. To substantiate this hypothesis, MRI images of the spinal cord of these patients were qualitatively reviewed. Indeed, a substantial amount of lesions in the spinal cord in these SPMS patients was found. Spinal lesion load has been shown to be more pronounced in SPMS than in RRMS patients and is positively correlated with the EDSS (39).

Previously published MRI-based classification schemes for MS patients commonly use either markers of inflammation like lesion load (40) or both inflammatory measures and brain atrophy (41, 42). The latter two scores by Bielekova et al. (41) and Tauhid et al. (42) define cut-off values for both lesion load and atrophy measures as the median in the examined cohort. These cut-offs, and therewith the scoring, will therefore largely depend on the cohort. The advantage of the grouping scheme presented here is the a priori defined cut-off for z-scores, that can be applied to MS cohorts without prior knowledge about the z-score distribution only requiring a cohort of healthy subjects. The pre-defined z-score cut-off of −1.96 represents an error probability of 2.5%. Future studies are needed to evaluate if this cut-off is appropriate. In order to increase the sensitivity, the cut-off could be set to a higher value, accepting a higher error rate for falsely including patients without atrophy. A stratification score for MS patients based on longitudinal measures of thalamus atrophy has also been proposed previously (43), but was not validated, yet. In order to measure thalamic atrophy longitudinally, the time interval between MRI examinations must be sufficiently large. The proposed grouping has the advantage of applicability at a single time point and may provide additional information for the clinician to complement the clinical representation at the first visit of the patient.

A limitation of this study is the small sample size in the subgroups. Especially the proportion of SPMS patients in this clinical outpatient cohort is small. If these patients are further subdivided according to the proposed grouping scheme, this yields quite small SPMS patient numbers per group. Therefore, Especially the results for the SPMS patients have to be interpreted with some caution. Another limitation of this study is that the individual treatment plans differed between included MS patients. Treatment might affect both the clinical scores and the extent of atrophy and might therefore distort the associations between z-scores and EDSS scores. However, the patient cohort reflects the heterogeneity of patients in clinical routine care.

## Conclusion

MRI-based brain volumetry at a single time point in combination with pre-defined cut-offs for z-scores enables reliable and standardized differentiation between MS patients in different stages of atrophy progression. With this method, we were able to show that thalamus atrophy is present in a significant subset of patients that do no exhibit whole brain atrophy yet. The proposed grouping can therefore provide information that goes beyond clinical assessments. This may aid identification of MS patients at risk of disease progression and thus might complement clinical evaluation of MS patients in clinical routine patient care.

## Author contributions

AR, LS, and GW made substantial contributions to the conception of the work, interpretation of data for the work, and revising it critically for important intellectual content. A-CO and RO made substantial contributions to the conception of the work, analysis, and interpretation of data for the work and drafting the work as well as revising it critically for important intellectual content. All authors gave final approval of the version to be published, and agreed to be accountable for all aspects of the work in ensuring that questions related to the accuracy or integrity of any part of the work are appropriately investigated and resolved.

### Conflict of interest statement

The authors declare that the research was conducted in the absence of any commercial or financial relationships that could be construed as a potential conflict of interest. The reviewer MS and handling Editor declared their shared affiliation.
