# Supplementary material for: MRI-Based Brain Volumetry at a Single Time Point Complements Clinical Evaluation of Patients With Multiple Sclerosis in an Outpatient Setting
Source: Front Neurol. 2018 Jul 25;9:545. doi: 10.3389/fneur.2018.00545 (PMC6095003; doi:10.3389/fneur.2018.00545)
Supplement: Supplementary file 1 [file Table_1.docx]

Supplementary Material

MRI-based brain volumetry at a single time point complements clinical evaluation of patients with multiple sclerosis in an outpatient setting

Alaleh Raji, Ann-Christin Ostwaldt*, Roland Opfer, Per Suppa, Lothar Spies, Gerhard Winkler

*** Correspondence:** Ann-Christin Ostwaldt: ann-christin.ostwaldt@jung-diagnostics.de

# Supplementary Table 1. Patient characteristics and volumetric results for the two MS patient populations (one scanned on the GE Signa 3 Tesla scanner system and one scanned on the Philips Achieva 3 Tesla scanner system) and the respective healthy controls. Brain volumes are uncorrected. All values are given as median and interquartile range or frequency and percentage. BP = brain parenchyma ; GM = gray matter; WM = white matter.

|  | **GE** |  | **Philips** |  |
| --- | --- | --- | --- | --- |
|  | **Patients** | **Controls** | **Patients** | **Controls** |
| **Sample size** | 130 | 34 | 55 | 50 |
| **Females** | 94 (72.3%) | 25 (73.5%) | 39 (70.9%) | 34 (68.0%) |
| **Age (years)** | 44 (33 - 51) | 44 (33-51) | 40 (32-49) | 53 (36-68) |
| **BP volume (ml)** | 1112 (1030-1212) | 1168 (1093-1234) | 1058 (979-1136) | 1057 (979-1155) |
| **GM volume (ml)** | 698 (635-750) | 717 (667-766) | 634 (587-681) | 613 (578-691) |
| **WM volume (ml)** | 423 (383-460) | 447 (417-482) | 412 (378-450) | 433 (394-472) |
| **Corpus callosum volume (ml)** | 18.7 (16.5-20.9) | 21.9 (20.3-23.6) | 18.6 (16.7-21.3) | 20.3 (18.5-22.9) |
| **Caudate nucleus volume (ml)** | 8.5 (7.5-9.1) | 9.5 (8.9-10.0) | 9.0 (7.8-9.4) | 9.0 (8.4-10.0) |
| **Thalamus volume (ml)** | 10.6 (9.5-11.8) | 12.4 (11.6-13.1) | 10.8 (9.8-11.6) | 11.4 (10.7-12.3) |
| **Putamen volume (ml)** | 19.2 (18.0-21.2) | 21.0 (19.6-22.5) | 20.2 (18.3-21.5) | 20.9 (19.0-22.7) |

**
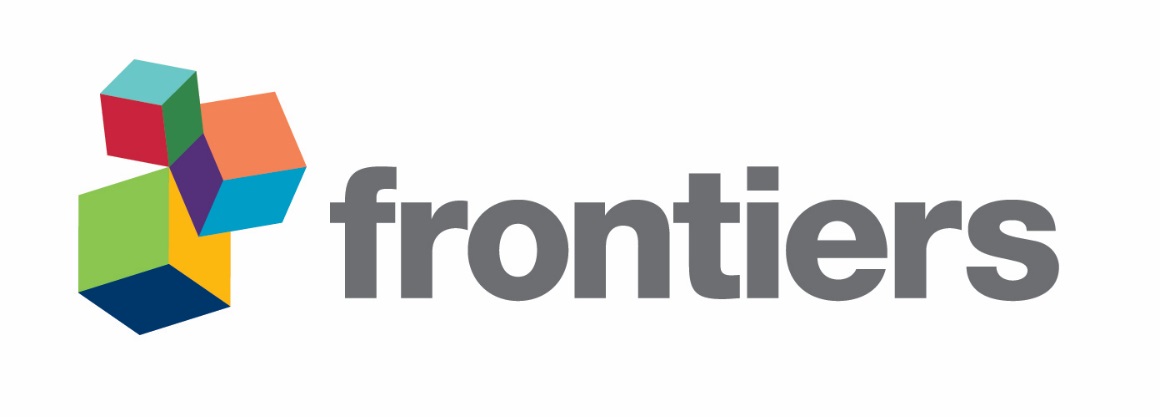
**

**
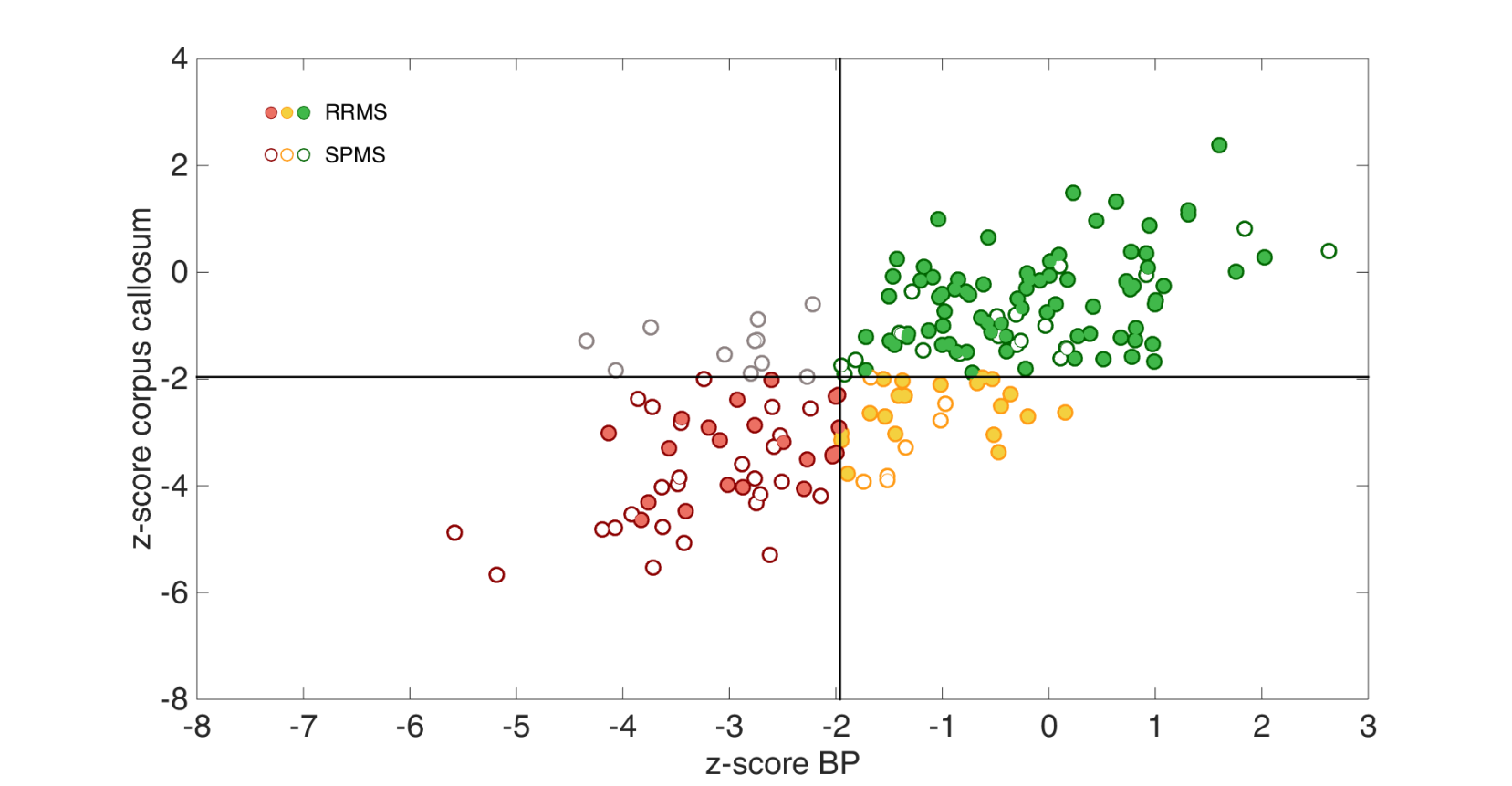
**

**Supplementary Figure 1.** Illustration of the grouping of the whole MS cohort according to corpus callosum and whole brain parenchyma (BP) atrophy. The association between z-scores for BP and z-scores for corpus callosum volumes are shown. In contrast to the grouping using thalamus z-scores (see Figure 2 for comparison), four groups are found when a cut-off of -1.96 is applied. Relapsing-remitting MS (RRMS) patients are marked with a filled circle in all groups, while secondary-progressive MS (SPMS) patients are marked with open circles.
